# OP62 Trends In Uncertainties And Outcomes Following An Assessment Of Cancer Topic Suitability For The Cancer Drugs Fund

**DOI:** 10.1017/S0266462325101190

**Published:** 2025-12-29

**Authors:** Sarika Paul, Leanne Wakefield, Catrin Austin, Milena Wobbe, Steve Norton, Robert Fernley, Steve Williamson

## Abstract

**Introduction:**

The Cancer Drugs Fund (CDF) is a type of managed entry agreement used in England to allow patient access while undertaking evidence generation followed by re-evaluation to address uncertainty. We assessed the impact of changes to the National Institute for Health and Care Excellence (NICE) health technology assessment (HTA) methods in 2022 plus the publication of a national commercial framework on recommendation outcomes.

**Methods:**

NICE staff, in conjunction with clinical stakeholders, assess all cancer medicines for their suitability for entry into the CDF. Cancer topics looked at by NICE from 2016 to 2024 were identified, and the type of evidence uncertainties and the final recommendation outcomes were recorded. Types of evidence uncertainty were assigned to one of nine different categories: survival data, relevant patient population, lack of relevant comparators, trial design, quality-of-life data, cost estimates, treatment duration, cost-effectiveness model, and adverse events. Recommendation outcomes included entry to the CDF, full reimbursement, reimbursement for optimized population, no reimbursement, or research only.

**Results:**

The types of uncertainties identified for appraisals using pre-2022 methods (n=103) and post-2022 methods (n=35) showed the same trends in most common uncertainties. Immature survival data was the most common, with lack of relevant comparators the second most common. There was an increase in the average number of uncertainties seen in post-2022 topics (2.05), compared with pre-2022 topics (1.75). The number of CDF recommendations has been decreasing in recent years, with a peak between 2017 and 2019. For appraisals that did not enter the CDF, the likelihood of a positive recommendation was lower in topics where entry to the CDF had been considered a possibility.

**Conclusions:**

There were no clear changes in trends of uncertainties seen in cancer topics following the 2022 methods changes, though there was a small increase in overall uncertainty. There was a decline in CDF entries, which correlates to payers offering a more flexible commercial environment giving alternative reimbursement options to evidence generation with re-evaluation via the CDF.

